# Health Tract, No. 190

**Published:** 1864-02

**Authors:** 


HEALTH TRACT, NO. 190.

### CHARMS.

EVEN in these late ages the horse-shoe is not unfrequently seen nailed over the door of the cabin or cottage, to "charm" away misfortune, or to "keep off" disease. There are intelligent men who have carried a buckeye in their "unmentionable" pockets for years, to "keep off" piles! Children can be found at school, any day, with little bags of brimstone attached to their necks by a string, to "keep off" some particular malady. There are many young gentlemen and ladies who have half a dozen "charms" attached to their watch-chains, it being a remnant of the ancient superstition. We give a pitying smile at the mention of these absurdities, for we know them to be unavailing. But there are "charms" against human ills which are powerful to save from physical, mental, and moral calamity!

Bearing about in one's heart the sweet memories of a mother's care, and affection, and fidelity, often has a resistless power, for many a year after that dear mother has found her resting-place in heaven, to restrain the wayward and the unsettled from rushing into the ways of wicked and abandoned men. John Randolph, of Roanoke, used to repeat in his later years, and always with quivering lips, that while he was quite a young man, in Paris, he was repeatedly on the point of plunging recklessly into the French infidelity which was so prevalent during the terrible "Revolution" of the time; but was as often restrained by the remembrance of that far-distant time, when yet in his infancy, his mother used to have him bend his knees before her, and, with his little hands in hers, taught him in sweet but tremulous tones to say nightly, "Our Father, who art," etc.

A Scotch mother, when her son, a lad of sixteen, was just about leaving for America, and she had no hope that she should ever meet him again, said to him: "Promise me, my son, that you will always respect the Sabbath day." "I will," said he. His first employer in New-York dismissed him because he refused to work on Sunday. But he soon found other employment, and is now a very rich man, an exemplary Christian, and an influential citizen.

Tens of thousands are there in this wide land who, by the "charm" of the temperance pledge, have gone out into the world, singly and alone, to battle with its snares, and temptations, and sin; they have been surrounded at every step by the great tempter, with the allurements of passion and pride; of sensual gratifications and of corrupting associations; but keeping their eye steadily fixed on the beautiful "pledge," to "touch not, taste not" the accursed thing, they have bravely come off conquerors, and to day stand in their might the pillars of society. Young gentlemen, and young ladies, too, make it your ambition to bear about with you "alway" the "charm" of the "pledge" of reverence for the Sabbath-day and the holy memories of a sainted mother's religious teachings, and you will pass safely to a ripe old age of happiness and health.

